# Biological role of NK cells and immunotherapeutic approaches in breast cancer

**DOI:** 10.3389/fimmu.2012.00375

**Published:** 2012-12-12

**Authors:** María P. Roberti, José Mordoh, Estrella M. Levy

**Affiliations:** ^1^Centro de Investigaciones Oncológicas, Fundación Cáncer FUCABuenos Aires, Argentina; ^2^Fundación Instituto Leloir, IIBBA-CONICETBuenos Aires, Argentina

**Keywords:** natural killer (NK) cells, breast cancer, immunotherapy, immunosuppression, monoclonal antibodies

## Abstract

In recent decades, tumor surveillance by the immune system and its impact on disease outcomes in cancer patients in general and in breast cancer (BC) patients in particular has been documented. Natural killer (NK) cells are central components of the innate immunity and existing data indicate that they play a role in preventing and controlling tumor growth and metastasis. Their biological significance was first recognized by their ability to exert direct cellular cytotoxicity without prior sensitization. This is important in tumors, as transforming events are likely to result in downregulation of self-ligands and expression of stress-induced ligands which can be recognized by NK cells. Their activation also leads to secretion of stimulatory cytokines which participate in cancer elimination by several direct mechanisms as well as by stimulating the adaptive immune system. In this regard, it was recently revealed a dendritic cell (DC)-NK-cell crosstalk which provides another novel pathway linking innate and adaptive immunity. In addition, NK cells are feasible targets of stimulation in immunotherapeutic approaches such as antibody-based strategies and adoptive cell transfer. Nevertheless, NK cells display impaired functionality and capability to infiltrate tumors in BC patients. This review compiles information about NK-cell biology in BC and the attempts which aim to manipulate them in novel therapeutic approaches in this pathology.

## Introduction

Natural killer (NK) cells have been first described as innate effector lymphocytes and their biological significance has been first recognized by their ability to exert direct cellular cytotoxicity without prior sensitization (Herberman et al., 1975; Kiessling et al., 1975). The missing-self hypothesis established by Klas Kärre and collaborators postulates that NK cells recognize and eliminate their target cells by sensing the loss of self-major histocompatibility complex (MHC) class I molecules (Karre et al., 1986; Ljunggren and Karre, 1990). As counterpart, when MHC class I inhibition is absent, the NK-cell must still be stimulated through activation receptors. For instance, the upregulation of stress signals switched on in cells under stress triggers NK-cell activation (Bauer et al., 1999). Alternatively, FcγIII receptor CD16 binds the Fc portion of antibodies and trigger an antibody-dependent cellular cytotoxicity (ADCC) on opsonized target cells (Titus et al., 1987). The regulation of effector function of NK cells then depends on the integration of these positive and negative signals sensed on target cells. This is particularly important in tumors, as the events leading to transformation are likely to result in downregulation of self-ligands and expression of stress-induced ligands by tumor cells which can then be recognized by NK cells (Bottino et al., 2005; Garrido et al., 2010).

## *In vivo* evidence of BC control by NK cells in a mouse model

Mouse models of human malignancies have contributed significantly to the understanding of disease pathogenesis as well as for preclinical therapeutic studies. Although several models mainly using conventional SCID mice are available, the major drawback is they still retain NK-cell, macrophage, complement, and dendritic cell (DC) activities. In contrast, NOD/SCID/γ^null^ (NSG) mice lack T, B, and NK cells which makes them a suitable model for tumor engraftment and to investigate the role of NK in tumor growth and metastasis (Ito et al., 2002). Using these models, the direct role of NK cells in tumor growth and metastasis was demonstrated *in vivo* by comparing NSG to conventional SCID mice. NSG mice inoculated with breast cancer (BC) cells were most efficient in the formation of large tumors within 2–3 weeks in all mice. Moreover, activated NK cells inhibited tumor formation and organ metastasis, suggesting that NK cells are responsible for inhibiting the formation of progressively growing rapid large tumors of BC cells in SCID mice (Dewan et al., 2007). A similar approach comparing wild type to NSG with BC cells showed that suppression of an Irf7-driven cluster of IFN-regulated genes is crucial to the establishment of bone metastases. Data showed that functional NK cells and CD8^+^ lymphocytes were both necessary for Irf7-induced and IFN-dependent immune activation to confer protection against metastasis but suggested that tumor immunosurveillance does not regulate the initiation of primary breast tumors. Furthermore, the clinical relevance of these findings was underscored in analyses of human primary tumors which revealed that high expression of the Irf7-regulated genes in patients with BC was associated with less relapses to bone (Bidwell et al., 2012).

## BC biology and NK cells

Human breast tumors can be categorized as luminal subtype A, luminal subtype B, HER-2^+^, basal subtype, normal breast-like, and the recently introduced Claudin-low subtype, based on their molecular characteristics (Sorlie, 2004; Prat et al., 2010). However, differential gene expression patterns in breast tumor stroma led to the identification of subtypes corresponding to good and poor-outcome BCs independently of molecular tumor type. Interestingly, tumor stroma samples from the good-outcome cluster overexpress a distinct set of immune-related genes, including T-cell and NK-cell markers indicative of a TH1-type immune response (granzyme A, CD52, CD247, and CD8A) (Finak et al., 2008). Although there is no evidence to date for an association between NK-cell infiltrate and clinical outcome in patients with BC, the expression of NK-cell ligands does play a crucial role in tumor immunoediting and concomitant immune escape in BC. This evidence arose from studies of prognostic value of non-classical HLA class I molecule expression in BC patients which showed that in tumors devoid of classical HLA class I expression, HLA-E and HLA-G expression were of statistically significant influence on outcome of BC patients independently of known clinicopathological parameters, with an almost three-times higher risk of relapse over time for patients with expression of HLA-E/G compared with patients with no expression of HLA-E/G (De Kruijf et al., 2010). Furthermore, an analysis of the clinical prognostic value of the activating NK-cell receptor NKG2D ligands MIC-AB and ULBP1-5 in early stage BC revealed that expression of MIC-AB and ULBP-2 results in a favorable outcome concerning relapse-free survival (De Kruijf et al., 2012). Microarray data for NK-cell ligand expression in primary breast tumors showed that the different subtypes express heterogeneous levels of inhibitory HLA members, while some patterns of ligand expression represented the different molecular subtypes, which are characterized by distinct genomic alterations and deriving from different precursor cells, except for NKG2D and DNAM-1 ligands which are expressed in virtually all breast tumors regardless molecular subtype (Mamessier et al., 2011a).

Besides the described alterations in tumor molecules, there are several studies that have shown that peripheral blood NK cells from BC patients have impaired functionality as well (Garner et al., 1983; Dewan et al., 2009; Mamessier et al., 2011b), contributing to tumor escape from NK-cell control. Indeed, tumor infiltrating NK cells display a striking phenotype with downregulation of activating receptors and the upregulation of inhibitory receptors, and evidence suggests that the tumor induces its own tolerance from NK-cell antitumor immunity (Mamessier et al., 2011a,b). Altogether, the analysis of these data revealed that, on the one hand, genomic variability and associated tumorigenic features observed in BC resulted of some successful immunoedition and, on the other hand, the existence of mechanisms possibly used by breast tumor cells to immunosubvert NK cells. From this, current efforts are focusing in identifying potential targets which would contribute to enhance anti-tumor efficiency (Mamessier et al., 2012).

Aside from direct tumor immune evasion, NK cells are the target of multiple indirect tumor escape mechanisms such as generation and maintenance of T regulatory cells (Ghiringhelli et al., 2006), and myeloid derived suppressor cells (Condamine and Gabrilovich, 2011). The pathological relevance of this tolerogenic and immunosuppressant environment has lately been brought up in tumor models and in patients with BC (Wolf et al., 2003; Sceneay et al., 2012). Consequently, further assessment of the presence of these mechanisms in BC patients should be considered for a better understanding of the complexity of immunoevasion and for designing successful NK-cell-based immunotherapy of BC.

## NK cells in BC therapeutics

### NK-cell-based immunotherapies

Manipulating the immune system for therapeutic benefit in cancer patients has been studied for well over 100 years. Targeted treatments and immunotherapy are two of the most promising approaches to cancer, and each could be improved by basic research on NK cells. NK cells-based immunotherapy in BC patients has two main approaches. One of them proposes the direct administration of these cells stimulated *ex vivo*, whereas the second one involve the administration of drugs, mainly cytokines, to stimulate NK cells in the patients themselves. Although, there have been great efforts to stimulate NK cells in adoptive therapies, remaining challenges are related to the maintenance of NK-cell stimulation over time and to obtain improved clinical outcomes. The first clinical trials were developed in the early nineties and continue to the present. The most important clinical trials using NK-cell-based immunotherapy are listed in Table 1.

**Table 1:** **Clinical studies and clinical trials in which NK-cell activity was assessed in BC patients**.

**Name**	**Patients (n° and scheme)**	**Aims (to test)**	**Results**		**References**
			**Clinical**	**NK-cell function**	
Polyadenylic-polyuridylic acid (PAPU) enhances the natural cell-mediated cytotoxicity in patients with BC undergoing mastectomy	PAPU or placebo was given *iv*. during the perioperative period (preoperative, days −1 and 0; postoperative, days 1, 3, 5, 7, and 14)	The effects of the biologic response modifier PAPU on natural cytotoxicity in patients with BC undergoing operation	PAPU prevented the decrease in the circulating number and cytotoxic activity of NK cells that occurred after operation and enhanced NK-cell cytotoxicity. This may have important implications for patients with BC undergoing major operation	Surgical procedures suppressed NK-cell cytotoxicity in the placebo group on post-operative days, whereas inhibition on post-operative day 2 failed to reach significance. PAPU abolished this immunosuppression after operation. The NK-cell activity was elevated when compared with the control group. Surgical procedures also reduced circulating NK-cell numbers during the first post-operative week in the placebo group; the decrease was statistically significant. The decrease in NK-cell numbers in the PAPU group was insignificant	Khan et al., 1995
Pilot immunoche-motherapy study with CMF, IL-2, and IFN-α	10 pts underwent alternating chemoim-munotherapy (cyclophosphamide + methotrexate + 5-ŕ fluorouracil + IL-2 + IFN) and CHT alone	Tolerability and the effects on host immunity of adjuvant CHT associated with IL-2 + IFNα in patients after surgery	9 pts completed 6 alternating cycles. 1 pt proved to have metastatic lesions after 4 cycles. The protocol was well tolerated, although leukopenia (CMF alone) and leukopenia with fever and moderate or minimal flu-like symptoms (CMF + IL-2 + IFN) were generally observed	Treatment with IL-2 facilitated complete recovery of white cell counts and NK-cell activity after the nadir on day 15	Tonini et al., 1998
Enhancement of the anti-tumor activity of a PB progenitor cell graft by mobilization with IL-2 + G-CSF in pts with advanced BC	43 women with stage IIIA/B or MBC underwent mobilization of PBPC with IL-2 s.c. for 14 days along with G-CSF for the latter 7 days. 15 women with stage IIIA/B or MBC underwent G-CSF mobilization alone and served as a control group	Dose-limiting toxicity and maximum tolerated dose of s.c. IL-2 with G-CSF for PBPC mobilization. Ability of mobilized SC to reconstitute hematopoiesis and the *in vitro* immunologic function of the graft in pts	IL-2 + G-CSF mobilization was safe, may lead to a more immunologically functional graft without impairing hematologic recovery. Limitations of this combined approach to SC mobilization include a decrease in the number of CD34^+^ cells mobilized with the combined cytokines and the short duration of the increased number of anti-tumor effector cells after transplant	There was no significant impact on time to engraftment of neutrophils or platelets using either mobilization regimen. The addition of IL-2 to mobilization increased the cytotoxicity of IL-2-activated mononuclear cells from the PBPC product against the BC cell target, MCF-7, and increased the percentage of NK cells and activated T cells in the PBPC product. The enhanced NK-cell number was sustained in the early posttransplant period	Burns et al., 2000
Posttransplant adoptive immuno-therapy with activated NK cells in pts with MBC	Cohort 1 (5 pts) received high-dose cyclophospha-mide, thiotepa, and carboplatin followed by PBSC infusion and GCSF. Cohort 2 (5 pts) received in addition IL-2 i.v. after PBSC infusion. In cohort 3 (5 pts), PBSC transplant was followed by infusion of autologous activated NK cells and IL-2	Effects of immunotherapy immediately after transplantation on engraftment and the associated toxicity	All pts has successful engraftment. All patients developed neutropenic fevers, but the overall toxicity associated with the infusion of IL-2 (cohort 2) or IL-2 plus activated NK cells (cohort 3) did not differ from that observed in cohort 1. CR were achieved in 1 pt in cohort 1, in 2 pts in cohort 2, and in 1 pt in cohort 3	Generation of activated NK cells was possible in all patients in cohort 3	de Magalhaes-Silverman et al., 2000
Pilot Trial of IL-2 + G-CSF	G-CSF (9 pts) vs. IL-2 and G-CSF (23 pts) in advanced pts receiving high-dose CHT with cyclophosphamide, thiotepa, and carboplatin	To defined immune, hematologic, and clinical effects of administration of IL-2 with G-CSF on mobilization of immune effectors into the SC graft of pts undergoing high-dose CHT	Mobilization of CD34^+^ SC seemed to be adversely affected. In those mobilized with IL-2 and G-CSF, post-SC immune reconstitution of antitumor immune effector cells was enhanced	G-CSF + IL-2 can enhance the number and function of antitumor effector cells in a mobilized autograft without impairing the hematologic engraftment. In particular, NK-cell number and activity was enhanced	Sosman et al., 2001
Administration of low-dose IL-2 + G-CSF/EPO early after autologous PBSC transplantation: effects on immune recovery and NK activity in a prospective study in women with BC and ovarian cancer	Post-PBSCT cytokine regimens (from day +1 to day +12) which consisted of G-CSF + EPO in 13 BC pts or G-CSF/EPO + IL-2 in 10 BC pts	The effects of low-dose IL-2 + G-CSF/EPO on post-PBSC transplantation immune-hematopoietic reconstitution and NK activity in pts with BC and ovarian cancer	Low-dose IL-2 can be safely administered in combination with GCSF/EPO early after PBSCT and that it exerts favorable effects on post-PBSCT myeloid reconstitution, but not on immune recovery	No significant difference between NK activity in the two groups, a significantly higher NK count was observed in G-CSF/EPO plus IL-2	Perillo et al., 2002
IL-2-based immunotherapy after autologous transplantation for lymphoma and BC induces immune activation and cytokine release: a phase I/II trial	Pts with relapsed lymphoma (*n* = 29) or MBC (*n* = 28) were enrolled. In part I of the study, 34 pts were enrolled at 3 dose levels of *ex vivo* IL-2-activated NK cells. Lymphaphereses were performed on days 28 and 42 of s.c. IL-2 administration. ON *ex vivo* IL-2-activated apheresis product was reinfused the following day. In part II, 23 pts were enrolled at 3 dose levels of supplemental i.v. IL-2 bolus infusions, during s.c. IL-2 administration	Safety, immune activating effects, and potential efficacy of i.v. infusion of *ex vivo* IL-2 activated NK cells (part I) or IL-2 boluses (part II) during daily s.c. IL-2 administration following hematopoietic recovery from autologous transplantation	Toxicities were generally mild, and no pt required hospitalization. The analysis demonstrated no improvement in disease outcomes of survival and relapse. With this dose and schedule of administration of IL-2, no improvement in pt disease outcomes was noted	Lytic function was markedly enhanced for fresh PBMC obtained 1 day postinfusion of either IL-2-activated cells or IL-2 boluses. IL-2 boluses transiently increased the levels of IL-6, IFN-γ, TNF-α, and IL1-β, with increases in IL-6 and IFN-γ being dose-dependent	Burns et al., 2003
Immune modulation and safety profile of adoptive immunotherapy using expanded autologous activated lymphocytes (EAAL) against advanced CA	19 pts (4 BC) with MTS tumors received EAAL therapy	Variation of peripheral lymphocyte phenotypes, the percentage of IFN-γ producing lymphocytes and the serum levels of IL-10 in the pts who received the adoptive cell therapy	There was no significant cell infusion toxicities (≥ grade II) observed. Anti-tumoral response showed 2 PRs (11.1%) and 10 stable diseases (SDs; 55.6%). There were no CR. Taken together, disease control rate (consisting of CR, PR, SD) was 66.7%. All the pts had progressive diseases. The median PFS was 5.0 months (95% confidence interval, 2.3–7.7) The median OS was 12.5 months (95% confidence interval, 7.8–16.2)	2–4 weeks after the administration of EAAL cells, the subsets of CD3^+^CD8^+^ T lymphocytes and CD3^−^CD56^+^ NK cells in the PB were increased significantly in comparison with those before the therapy. Moreover, the percentage of IFN-γ producing cells of the CD3^+^, CD8^+^ and CD3^−^ subsets after infusion were all increased significantly, which indicated that EAAL therapy was able to enrich the potentially anti-tumor cytotoxic PB lymphocytes	Sun et al., 2011
A phase II study of allogeneic NK-cell therapy to treat pts with recurrent ovarian and BC	20 (14 ovarian, 6 BC) pts underwent a lymphodepleting regimen: fludarabine, cyclophosphamide, and, in 7 pts, 200 cGy total body irradiation (TBI). A haplo-identical NK-cell product IL-2 preactivated was infused s.c. IL-2 was given after infusion to promote expansion	Tumor response and *in vivo* expansion of allogeneic NK cells in recurrent ovarian and BC	Adoptive transfer of haplo-identical NK cells after lymphodepleting CHT is associated with transient donor chimerism and may be limited by reconstituting recipient Treg cells. Strategies to augment *in vivo* NK-cell persistence and expansion are needed	During the lymphopenic window induced by the preparative regimen, there was evidence of transient donor NK-cell persistence, but by day 14 pts had reconstituted with host T cells, suggesting that suppressive factors induced in the tumor microenvironment may be inhibiting donor NK-cell expansion. Cyclophosphamide and fludarabine alone, or with 200 cGy TBI, were inadequate to promote NK-cell expansion	Geller et al., 2011
Phase I Trial of 2B1, a Bispecific M Ab Targeting c-erbB-2 and FcyRIII	15 pts with c-erbB-2-overexpressing tumors were treated with i.v. infusions of 2B1 6 days single course of treatment. pts were treated with different doses	(a) to identify the toxicity and maximally tolerated dose of 2B1 using a daily 1 h infusion schedule; (b) to examine the pharmacokinetics of i.v. infused 2B1; (c) to examine the bio-distribution in PB of 2B1; and (d) to determine the immunogenicity of 2B1	The principal non-dose-limiting transient toxicities were fevers, rigors, nausea, vomiting, and leukopenia. Thrombocytopenia was observed in 2 pts who had received extensive prior myelosuppressive CHT. Brisk human anti-mouse Ab responses were induced in 14 of 15 pts. Several minor clinical responses were observed, with reductions in the thickness of chest wall disease in 1 pt with disseminated BC. Resolution of pleural effusions and ascites, were noted in 2 pts with MTS colon CA., and 1 of 2 liver MTS resolved in a pt with metastatic colon CA	Murine Ab was detectable in serum following 2B1 administration, and its bispecific binding properties were retained. The pharmacokinetics of this munne Ab were variable and best described by non-linear kinetics with an average t_1/2_ of 20 h. Murine Ab bound extensively to all neutrophils and to a proportion of monocytes and lymphocytes. The initial 2B1 treatment induced more than 100-fold increases in circulating levels of TNF-α, IL-6, and IL-8 and lesser rises in GMCSF factor and IFN-γ	Weiner et al., 1995
A Phase I Trial of Escalating Doses of TRZ Combined with Daily Sc IL-2	Eligible pts had non-hematological malignancies for which standard therapy did not exist or was no longer effective and HER2 overexpressing tumors. IL-2 was initially administered at a dose of 1.25 million IU/m2 (low dose) s.c. Daily. TRZ was administered i.v. just before the first intermediate IL-2 dose and was escalated in cohorts of 6 or more pts	The toxicity of escalating doses of TRZ when combined with a fixed dose regimen of IL-2	45 pts were treated. Dose-related toxicity from TRZ was not observed. IL-2-related toxicities such as fever, chills, and fatigue were less common with the reduced doses of IL-2. There were two grade 3 and three grade 4 pulmonary reactions. 4 major responses were observed, all in BC pts treated with TRZ doses of at least 4.0 mg/kg	Although IL-2 produced expansion of NK-cell subsets, there was no correlation between *in vitro* cytotoxicity and clinical response	Fleming et al., 2002
TRZ and IL-2 in HER2-+ MBC: a pilot study	10 pts with HER2-overexpressing MBC were treated with IL-2 s.c. for 7 weeks and TRZ for 6 weeks	To test if IL-2 can increase efficacy, be safely given, and avoid the use of CHT, when added to TRZ	10 women received a total of 12 cycles of therapy (each cycle lasted 7 weeks). No significant toxicities were seen, and 1 pt required an IL-2 dose reduction. Among the evaluable pts, there were one PR, 5 cases of SD, and 4 of PD	*In vitro* immune assays showed NK-cell expansion and TRZ-mediated increased NK-cell killing of BC targets (ADCC) in a HER2-specific manner but did not correlate with clinical responses	Repka et al., 2003
A phase I study of IL-12 with TRZ in pts with Her2-over-expressing malignancies	Pts with MTS HER2+ malignancies received TRZ on day 1 of each weekly cycle. Beginning in week 3, pts also received i.v. injections of IL-12. The IL-12 component was dose-escalated within cohorts of 3 pts. Correlative assays were conducted using serum samples and PBMC obtained during the course of therapy	Safety and optimal biological dose of IL-12 when given in combination with TRZ	15 pts were treated. The regimen was well tolerated with IL-12-induced grade 1 nausea and grade 2 fatigue predominating. Evaluation of dose-limiting toxicity and biological end points suggested that the 300 ng/kg dose was both the maximally tolerated dose and the optimal biological dose of IL-12 for use in combination with TRZ. 2 pts with HER2 3+ BC experienced grade 1 asymptomatic decreases in left ventricular ejection fraction of 12% and 19% after 3 and 10 months of therapy. There was 1 CR in a pt with HER2 3+ MBC to the axillary, mediastinal, and supraclavicular nodes, and 2 pts with stabilization of bone disease lasting 10 months and >12 months	Correlative assays showed sustained production of IFNγ by NK cells only in those pts experiencing a clinical response or stabilization of disease. Elevated serum levels of MIP-1, TNF-α, and the antiangiogenic factors IP-10 and MIG were also observed in these pts. Pts genotyping suggested that a specific IFN-γ gene polymorphism might have been associated with increased IFN-γ production. The ability of pts PBMC to conduct ADCC against tumor targets *in vitro* did not correlate with clinical response or dose of IL-12	Parihar et al., 2004
Phase 1B/2 Trial of 2B1 Bispecific Murine (mAb) in MBC (E3194)	20 women with MBC	Redefined the maximally tolerated dose of this Ab for this selected PT population	Objective antitumor responses were not seen	2B1 therapy-induced adaptive immune responses to both intracellular and extracellular domains of HER2/neu	Borghaei et al., 2007
A phase I trial of PCTX and TRZ in combination with IL-12 in patients with HER2/neu-expressing malignancies	16 pts had more than one MTS site. 16 of 21 pts had received at least one prior regimen of systemic CHT, including 4 who had previously received PCTX. 9 pts (43%) had 3+ expression of HER2/neu, 12 (57%) had 2+ expression. 7 pts had MBC, but 14 pts with other malignancies were also accrued	Safety profile of IL-12 in combination with TRZ and PCTX to pts with metastatic HER2-overexpressing ca	There was 1 complete response in a PT with BC, partial responses in 4 pts (BC, 2; esophageal, 2), and stabilization of disease lasting 3 months or greater in 6 other pts. All but 1 response in pts with HER2 3+ disease. 2 pts completed 1 year of therapy. 10 pts had progressive disease. IL-12 + TRZ and PCTX exhibits an acceptable toxicity profile and has activity in pts with HER2-overexpressing CA	Increased activation of extracellular signal-regulated kinase in PBMC and increased levels of IFN-γ and several chemokines in pts with clinical benefit (CR, PR, but not in patients with PD)	Bekaii-Saab et al., 2009
A phase II trial of TRZ with low-dose IL-2 in pts with MBC who have previously failed TRZ	Pts received TRZ with daily low-dose IL-2 or intermediate-dose IL-2	IL-2 effect on the anti-tumor effects of TRZ in MBC in pts who had progressed on or within 12 months of receiving a TRZ-containing regimen. Secondary objectives were to measure ADCC against HER2 over-expressing cells, and to measure serum cytokines	The median number of treatment cycles was four (range 1–23) and the treatment was well tolerated. There were no objective responses. In TRZ-refractory pts adding IL-2 did not produce responses	NK cells were not expanded and ADCC was not enhanced. 8 (62%) pts had a 2-fold or higher increase in mRNA for IFN-γ 2 (15%) pts had elevated serum levels of IFN-γ and 12 (92%) had increases angiogenic MIG and IP-10. The lack of NK-cell expansion may explain the absence of clinical benefit	Mani et al., 2009

ADCC, Antibody-Dependent Cellular Cytotoxicity; BC, Breast cancer; CHT, Chemotherapy; CR, Complete response; i.v., intra venous; IFN-γ, Interferon gamma; IL, Interleukin; IP-10, IFN, inducible protein 10; mAb, Monoclonal Antibody; MBC, Metastatic BC; MIG, Monokine-induced by IFN-γ ; MIP-1, Macrophage Inflammatory prot 1; MTS, Metastatic; PB, Peripheral blood; PBMC, Peripheral blood Mononuclear Cells; PCTX, Paclitaxel; PD, Progressive disease; PR, Partial response; pts, Patients; s.c., Subcutaneously; SC, Stem Cells; SD, stable disease; TRZ, Trastuzumab.

### Therapeutic antibodies and NK cells

Targeting therapies with monoclonal antibodies (mAbs) are also important therapeutic strategies for cancer. The mAb trastuzumab has been approved for clinical use and has become a mainstay for HER2-positive BC (Slamon et al., 2001). Clinical efficacy is believed to rely mainly on the interference with the HER2 oncogenic signaling. In addition, one of the potential mechanisms of action of trastuzumab is NK-cell-mediated ADCC of the HER2-positve target cells. In this sense, the HER2 subtype is perhaps today the BC subtype in which we can explore the direct activity of NK cells in therapeutics. Trastuzumab-mediated ADCC potency displayed by peripheral blood NK cells was shown to be correlated with the short-term response to treatment in metastatic BC patients (Beano et al., 2008). Further examination of factors affecting ADCC intensity and variability showed that the ability to develop ADCC in patients was significantly dependent on the quantity of CD16^+^CD56^+^ lymphocytes among Peripheral blood Mononuclear Cell (PBMC). However, when quantity was normalized to target cell number, there was a relationship between CD16-158V/F polymorphism and the killing efficiency of CD16^+^CD56^+^ cells (Varchetta et al., 2007). However, the largest retrospective analysis to date evaluating an association between FCGR3A/2A genotypes and clinical outcome in trastuzumab-treated HER2-positive BC in the adjuvant setting showed no statistically significant correlation between FCGR3A and FCGR2A genotypes and disease-free survival in a cohort of 1286 patients treated with trastuzumab-based therapy in early BC nor in progression-free survival in 53 women treated with trastuzumab-based therapy for metastatic BC (Hurvitz et al., 2012). While these data do not entirely dismiss the possibility that trastuzumab triggers ADCC as one of its mechanisms of action, it does suggest that any differences in Fc-FcγR affinity attributed to the polymorphisms tested does not result in detectable differences in clinical outcome. In fact, other evidence supporting the ADCC role in trastuzumab therapeutic action was also demonstrated. E-cadherin-expressing HER2-overexpressing BC cells were induced to treatment resistance toward trastuzumab, because ADCC activity was inhibited by interaction with E-cadherin and KLRG1 (Yamauchi et al., 2011). Recently, a novel N-terminal fragment of HER2 was characterized (H2NTF) and found to be frequently expressed in breast tumor samples at variable levels. H2NTF is devoid of the tyrosine kinase domain but it readily interacts with full-length HER2 and other HER receptors. Therefore, H2NTF acts as a dominant negative, attenuating the signaling triggered by full-length HER receptors. Expression of H2NTF results in resistance to the treatment with low concentrations of trastuzumab *in vitro*. However, cells expressing H2NTF and non-expressing cells have similar sensitivity to trastuzumab *in vivo*, indicating that H2NTF/trastuzumab complexes trigger ADCC (Morancho et al., 2012). Given the significance of the ADCC as antitumor function, it is reasonable to think that the efficiency of the host immune system could influence the responses to current therapy regimens. Recently, an immunophenotypic profiling of circulating and intratumor immune cells determined that compared to HER2-cases, patients with HER2-overexpressing locally advanced BC show a more limited tumor-related immune suppression (Muraro et al., 2011). This may account for the clinical benefit achieved in this subset of patients with the use of drugs acting through, but also promoting, immune-mediated effects.

Based on its ADCC mechanism, the anti-tumor efficacy of trastuzumab can be enhanced by means of combination with other immunomodulatory approaches such as chemotherapy, radiotherapy, targeted therapy agents, vaccines, or other immunomodulators. Table 1 also shows clinical trials that demonstrate the use and effectiveness of trastuzumab combined with different agents.

Experience with trastuzumab arouses the hope that mAbs could be used in other BC subtypes. Particularly important is the need for target therapies in basal BC as these patients are not likely to benefit from anti-estrogen or anti-HER2 therapy, and first-line treatment usually consists of conventional cytotoxic chemotherapy. Although basal BCs are quite sensitive to chemotherapeutic regimens in the preoperative setting, they are nevertheless associated with poor long-term outcomes (Liedtke et al., 2008). Nevertheless, better prognosis groups of basal BCs were identified on the basis of the expression of genes involved in antitumor immunity (Teschendorff et al., 2007; Sabatier et al., 2011a,b). As epidermal growth factor receptor (EGFR) is a targetable receptor frequently overexpressed in basal BC, currently cetuximab, a mAb that binds EGFR, is being tested for efficacy in TNBC (Carey et al., 2012) and it was also shown *in vitro* that it triggers NK-cell-mediated ADCC (Roberti et al., 2011).

### NK-cell—DC crosstalk

Besides effector functions for tumor elimination and cytokine production, NK cells have mechanisms for stimulating the adaptive immune system, thus, being instrumental for enhancing antigen processing and presentation. Recent studies illustrate the potential of NK cells to increase the immunostimulatory capacity of DCs. It was demonstrated that NK cells can efficiently promote maturation and differentiation of DCs toward a Th1 profile (Mailliard et al., 2003). Hence, by inducing DC activation, NK-cell activation induced by tumor cells can indirectly promote antitumoral T-cell responses. Reciprocally, activated DCs induce potent NK-cell activation. Thus, NK-cell/DC crosstalk coordinates innate and adaptive immune responses influencing the quality and strength of the global immune response (Moretta et al., 2006). In line with this, it was recently identified that the interaction of cetuximab with EGFR on BC tumor cells and FcγR IIIa on NK cells enhances cross-presentation of tumor antigens, such as EGFR, by DC to cytotoxic T lymphocytes (Lee et al., 2011). Regarding the evidence in clinical studies, correlation of clinical responses in DC vaccine treated patients was reported. A phase I/II trial of an autologous DC vaccine combined with IL-2 induced specific immunity against introduced antigens and modifications of immunological parameters such as antigen-specific immune induction and NK-cell activation along with reduction of inhibitory immunity in vaccinated patients (Baek et al., 2011). However, the effect on the NK-cell population might be due to the IL-2 administration. In a more recent study of only autologous DC-based immunotherapy in treatment of stage II/IIIA BC patients data supports a correlation between positive immune responses induced by DC vaccination and improved long-term progress-free patient survival. DC vaccination was capable of promoting secretion of Th1 cytokines, as well as of significantly increased the number of peripheral blood NK cells and leding to tumor-specific CD8^+^ T-cell expansion and further differentiation into IFN-γ-producing effector cells (Qi et al., 2012).

## Perspectives

The compiling evidence of NK-cell biology in BC sheds light on the mechanisms involved in NK-cell tumor evasion and highlights the role of NK cells in the control of invasive breast tumors. However, regarding tumor strategies to overcome its elimination by innate immunity, it stresses the necessity of attempts which aim not only to stimulate NK cells directly but to target the tumor counterattack mechanisms in order to obtain clinical success in novel therapeutic approaches to battle this complex pathology.

### Conflict of interest statement

The authors declare that the research was conducted in the absence of any commercial or financial relationships that could be construed as a potential conflict of interest.
